# Supplementary material for: Magnetically-induced ferroelectricity in the (ND4)2[FeCl5(D2O)] molecular compound
Source: Sci Rep. 2015 Sep 29;5:14475. doi: 10.1038/srep14475 (PMC4586439; doi:10.1038/srep14475)
Supplement: Supplementary Information [file srep14475-s1.pdf]

Supporting information for:

Magnetically-induced ferroelectricity in the $(ND_4)_2[FeCl_5{\cdot}D_2O]$ molecular compound.

José Alberto Rodríguez-Velamazán,[1,2] Óscar Fabelo,[2*] Ángel Millán,[1] Javier Campo,[1] Roger Johnson,[3] Laurent Chapon.[2*]

[1] Instituto de Ciencia de Materiales de Aragón (ICMA), CSIC – Universidad de Zaragoza, 50009 Zaragoza, Spain.

[2] Institut Laue-Langevin, 38042 Grenoble Cedex 9, France.

[3] Univ Oxford, Dept Phys, Clarendon Lab, Oxford OX1 3PU, England

Table S1. Experimental parameters and main structural crystallographic data for the studied compounds determined from single crystal X-ray diffraction.

Formula	$[NH_4]_2[Fe^{III}Cl_5(H_2O)]$	
Empirical Formula	$Cl_5H_{10}FeN_2$	
Mr (g·mol-1)	287.20	
Temperature (K)	293(2)	50(2)
λ(Å)	0.71073	0.71073
Crystal system	Orthorombic	Orthorombic
Space group (No.)	*Pnma* (62)	*Pnma* (62)
Crystal size (mm)	0.08 × 0.08 × 0.06	0.08 × 0.08 × 0.06
a (Å)	13.728(5)	13.5088(6)
b (Å)	9.934(5)	9.9408(4)
c (Å)	7.040(5)	6.9070(4)
α(°)	90.00	90.00
β(°)	90.00	90.00
γ(°)	90.00	90.00
V (Å^3)	960.0(9)	927.53(8)
Z	4	4
ρ_c (g·cm^{-3})	1.987	2.057
Meas. Reflections/ (Rint)	26820/(0.0442)	16496/(0.0931)
Indep. ref. [I > 2σ(I)]	943	923
Parameters/ restraints.	69/ 4*	69 / 4*
Hydrogen treatment	Refall	Refall
Goodness of fit	1.139	1.132
Final R indices [I > 2σ(I)]: R_1 / wR_2	0.0387/ 0.0593	0.0554/0.0803
R indices (all data): R_1 / wR_2	0.0768/0.0694	0.1011/0.0906

*The hydrogen atoms where refined using a DFIX command with a N-H bond distance of 0.90(3)

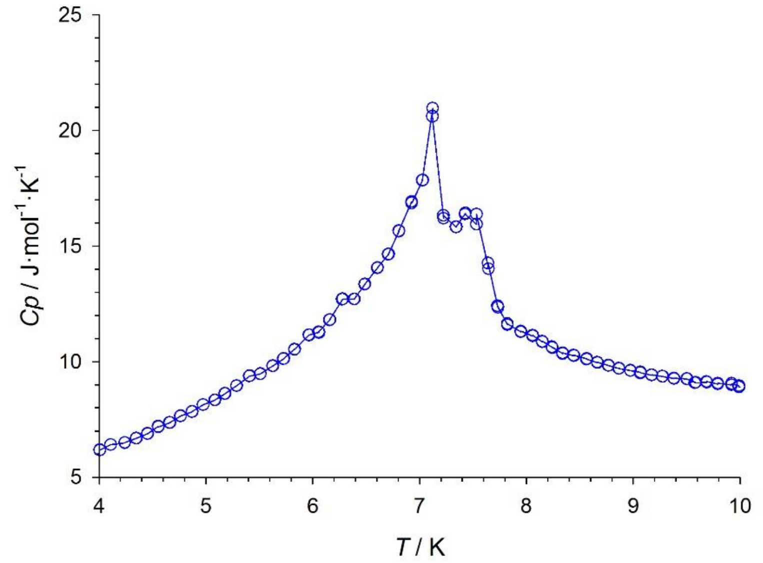

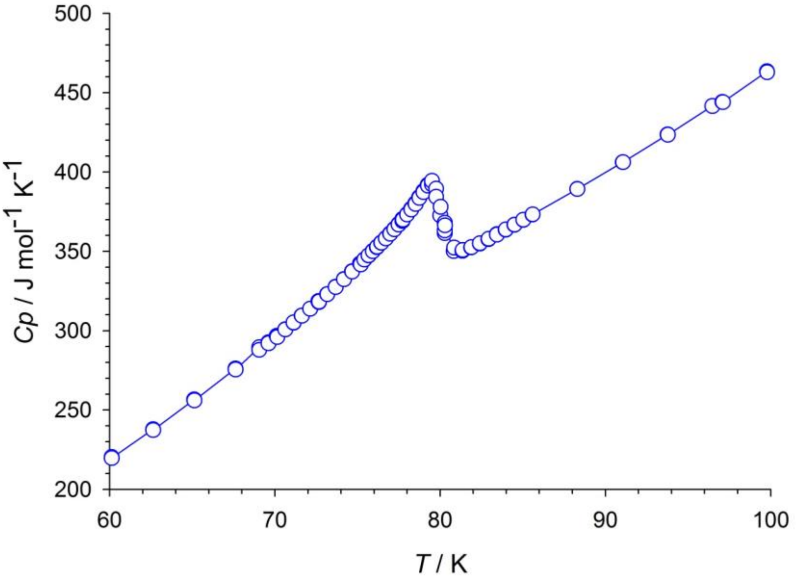

Figure S1. (Top) Detail of the heat capacity curve of $(ND_4)_2[FeCl_5(D_2O)]$ compound in the low temperature range. The two different phase transitions observed (long range magnetic ordering at ca 7.4 K and ferroelectric ordering at ca 7.1 K) are in agreement with those previously characterized for the non-deuterated compound. (Bottom) Detail of the heat capacity curve of $(ND_4)_2[FeCl_5(D_2O)]$ compound in the region of the high-temperature transition. The phase transition appears slightly shifted of ca. 1K with respect to the hydrogenated form.

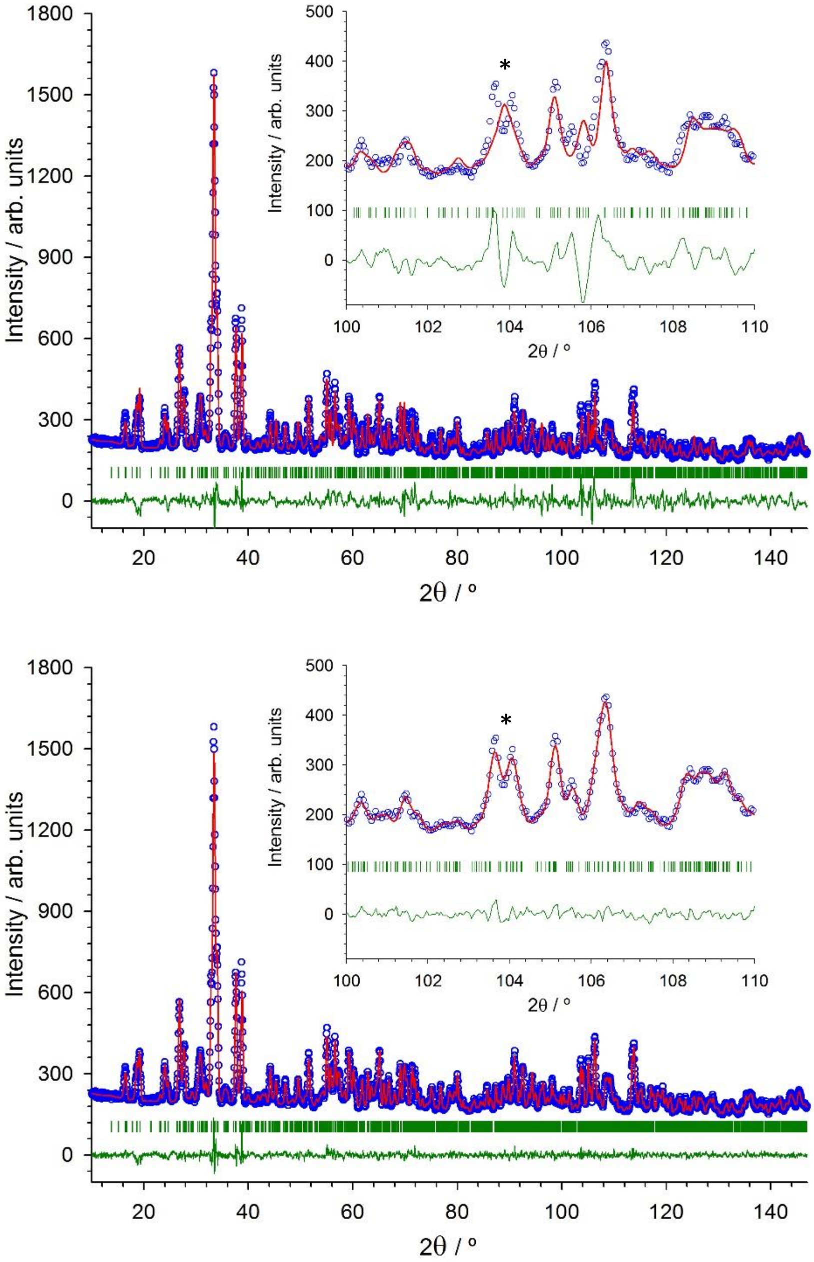

Figure S2. Neutron diffraction pattern of $(ND_4)_2[FeCl_5(D_2O)]$ compound collected at 45K using D2B high resolution instrument with λ= 1.5942 Å. The experimental data have been represented as blue circles, the calculated curve as solid red line and the difference between them as solid green line. The green vertical lines represent the Bragg positions for the selected space group. The Rietveld refinements were done in the space group *Pnma* (up) and in $P112_1/a$ (bottom), the insets show the splitting due to the nuclear phase transition from orthorhombic to monoclinic system. For example, in the zone marked with a star, the *Pnma* fit produces a calculated peak that corresponds to the contribution of (12 4 1) and (5 8 3) reflections, at 2θ= 103.57^o and 103.66^o respectively, while in the $P112_1/a$ fit, these two reflections are split in (-12 4 1) and (-5 8 3), at 2θ= 103.36^o and 103.51^o respectively, on one side, and (12 4 1) and (5 8 3), at 2θ= 103.77^o and 103.85^o, on the other side, giving rise to the correct fit of the experimental data.

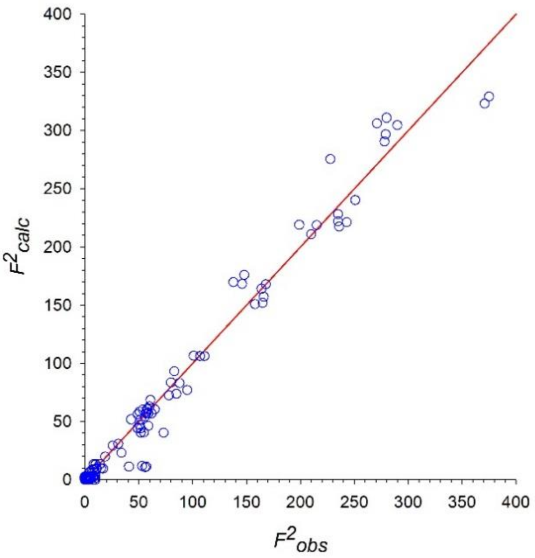

Figure S3. Squared magnetic structure factors observed *versus* calculated for D9 instrument at 2K. The refinement has been done with 127 magnetic reflections, with an agreement factor of R_F = 12.8%.
